# Cytomegalovirus Viremia Is Strongly Associated With 6-Month Mortality Among Adults Living With Advanced HIV Disease: A Prospective Cohort Study in South Africa

**DOI:** 10.1093/ofid/ofag615

**Published:** 2026-09-30

**Authors:** Jayne Ellis, Nelesh P Govender, Nei-yuan Hsiao, Tanvier Omar, Carolina Nel, Ahmad Haeri Mazanderani, Caroline T Tiemessen, Thomas S Harrison, Joseph N Jarvis, Rachel M Wake

**Affiliations:** Department of Clinical Research, Faculty of Infectious and Tropical Diseases, London School of Hygiene and Tropical Medicine, London, UK; Wits Mycology Division, School of Pathology, Faculty of Health Sciences, University of the Witwatersrand, Johannesburg, South Africa; Department of Pathology, University of Cape Town, Cape Town, South Africa; Institute for Infection and Immunity, City St George's University of London, London, UK; MRC Centre for Medical Mycology, University of Exeter, Exeter, UK; Department of Pathology, University of Cape Town, Cape Town, South Africa; Department of Anatomical Pathology, School of Pathology, Faculty of Health Sciences, University of the Witwatersrand, Johannesburg, South Africa; Wits Mycology Division, School of Pathology, Faculty of Health Sciences, University of the Witwatersrand, Johannesburg, South Africa; Centre for HIV and STIs, National Institute for Communicable Diseases, A Division of the National Health Laboratory Service, Johannesburg, South Africa; Faculty of Health Sciences, University of the Witwatersrand, Johannesburg, South Africa; Centre for HIV and STIs, National Institute for Communicable Diseases, A Division of the National Health Laboratory Service, Johannesburg, South Africa; Faculty of Health Sciences, University of the Witwatersrand, Johannesburg, South Africa; Institute for Infection and Immunity, City St George's University of London, London, UK; MRC Centre for Medical Mycology, University of Exeter, Exeter, UK; Infection Clinical Academic Group, St George's Hospital NHS Trust, London, UK; Department of Clinical Research, Faculty of Infectious and Tropical Diseases, London School of Hygiene and Tropical Medicine, London, UK; Botswana Harvard Health Partnership, Gaborone, Botswana; Wits Mycology Division, School of Pathology, Faculty of Health Sciences, University of the Witwatersrand, Johannesburg, South Africa; Institute for Infection and Immunity, City St George's University of London, London, UK; Infection Clinical Academic Group, St George's Hospital NHS Trust, London, UK

**Keywords:** AIDS, cytomegalovirus, HIV, minimally invasive autopsy

## Abstract

Cytomegalovirus viremia was detected in 59% (86/146) of adults with very advanced human immunodeficiency virus (HIV) disease (CD4 < 100 cells/µL) attending care in South Africa and was strongly associated with increased 6-month mortality after adjustment for CD4 count and antiretroviral therapy (adjusted odds ratio 4.02, 95% CI 1.27–12.7).

## BACKGROUND

More than 4 million people are living with advanced human immunodeficiency virus (HIV)-1 disease (AHD; CD4 < 200 cells/μL or a World Health Organization stage 3 or 4 disease [1]). Despite access to antiretroviral therapy (ART), and screening and preemptive treatment programs for tuberculosis and cryptococcosis, mortality among outpatients living with AHD remains high; 15% of individuals die within a year of ART initiation [2, 3]. The causes of this excess mortality are poorly understood, and novel strategies to improve survival are needed.

Cytomegalovirus (CMV) is a ubiquitous human herpesvirus. In low- and middle-income settings, most children acquire both infections in the first few years of life. In a study of 2174 Ugandan children and adults, 95% of participants were CMV seropositive by 5 years of age [4]. Following acute infection, CMV can sustain lifelong latency within the host with potential for intermittent reactivations. Due to profound immunosuppression and immune dysregulation, reactivation of CMV is common among people with AHD [5–8]. Since the onset of the HIV epidemic, studies have consistently shown that CMV viremia is associated with worse outcomes in the context of AHD, including an increased risk of AIDS-defining events, tuberculosis, and death [9–11]. In the contemporary dolutegravir-based ART era, we have demonstrated that CMV viremia (without CMV end-organ disease) is common (∼50% prevalence) and is associated with 2-fold higher mortality among hospitalized adults with AHD and cryptococcal meningitis [7, 12]. We hypothesized that CMV viremia is also prevalent and may contribute to mortality among outpatient populations living with AHD.

We conducted an observational cohort study among adults with CD4 counts < 100 cells/mm^3^ to determine the prevalence of CMV viremia and assess its associations with opportunistic infections and mortality.

## METHODS

This was a post hoc nested cohort study of the CICAS study [13]. Between June 2015 and October 2017, we enrolled adults (≥18 years) with CD4 count < 100 cells/mm^3^ attending HIV care following cryptococcal antigen (CrAg) screening, with and without cryptococcal antigenemia (ratio 1:2) at 2 hospitals in South Africa: Helen Joseph and Tambo Memorial hospitals, Johannesburg. Patients were excluded if they had symptoms of cryptococcal meningitis or if they were receiving treatment for cryptococcal meningitis at baseline.

At study enrollment, demographic data, clinical characteristics, and clinical samples for tuberculosis, cryptococcosis, and histoplasmosis screening were collected (Supplementary Methods 1). Opportunistic infections were treated in accordance with local guidelines. Study participants were followed-up via telephone on 3 occasions over 6 months to ascertain hospitalization events, diagnosis of incident opportunistic infections, and survival and cause of death, including minimally invasive autopsy (MIA) for a subset of deceased participants. Minimally invasive autopsy procedures were similar to those described in previous MIA studies [14, 15] (Supplementary Methods 2).

All participants with a stored baseline plasma sample available were included in this substudy; cryopreserved samples were tested by batch CMV viral load assay (Abbott RealTime CMV, Abbott Molecular Inc., Abbott Park, IL, USA, limit of detection 50 IU/mL) at the University of Cape Town. Laboratory-based investigators were blinded to participant data. Cytomegalovirus viremia was defined as detectable CMV deoxyribonucleic acid (DNA) in plasma.

Baseline characteristics were summarized as counts and percentages or medians (with interquartile ranges [IQRs]). The proportion of participants with CMV viremia with 2-sided 95% CI was determined. Baseline variables were compared by CMV viremia status, using the χ² test for proportions, and the Mann–Whitney *U*-test or Student's *t*-test for continuous data.

Logistic regression models were used to investigate associations between CMV viremia and 6-month mortality (Supplementary Figure 1). To explore a potential dose–response relationship between CMV viremia and mortality, we dichotomized participants with CMV viremia into “low” and “high” level groups using a threshold of ≥1000 CMV IU/mL (a preemptive treatment threshold frequently used in hemopoietic transplant cohorts [16]). In the adjusted regression models, age, sex, baseline CD4 count, and ART status at the time of enrollment were included a priori. All other covariates were considered as potential confounders, other than those considered to be potentially on the causal pathway between the coinfection and mortality and hence not included in the regression models (Supplementary Figure 1).

A likelihood ratio test was conducted looking for an interaction between ART status and CrAg status with CMV viremia status and mortality, using an adjusted logistic regression model. Kaplan–Meier curves were plotted to 6 months stratified by CMV viremia status; hazard ratios were estimated using a Cox proportional hazards model.

## PATIENT CONSENT STATEMENT AND REGULATORY APPROVALS

Institutional Review Boards approvals were obtained from the University of the Witwatersrand and the London School of Hygiene & Tropical Medicine. All participants provided written informed consent, including premortem consent for autopsy.

## RESULTS

We performed CMV viral load testing for 146 participants. The median age was 39 (IQR 33–48) years, and 50% (73/146) were male. At baseline, the median CD4 count was 32 (IQR 10–60) cells/mm^3^, and 20% (29/146) were receiving ART. Of included participants, 33% (48/146) were CrAg positive in blood.

Baseline CMV viremia was detected in 59% (86/146, 95%CI 50–67%), with a median viral load of 366 (IQR 171–1187) IU/mL. Of those with CMV viremia, the majority (72%, 62/86) had a CMV viral load of <1000 CMV IU/mL. Participants with CMV viremia had lower median baseline CD4 counts versus those without CMV viremia (26 vs 38 cells/μL, *P* = .04). There was no evidence that CMV viremia was associated with diagnosis of cryptococcal antigenemia or concurrent tuberculosis (TB) disease at baseline (Supplementary Table 1).

Overall, all-cause 6-month mortality was 18% (26/146); mortality was significantly higher in those with CMV viremia (26%, 22/86) compared to those without (7%, 4/60, *P* = .003). Participants with CMV viremia had greater than 4 times the odds of 6-month mortality (OR 4.81, 95% CI 1.56–14.8, *P* = .006) compared to those without CMV viremia. After adjustment for age, sex, CD4 count, and baseline ART status, CMV viremia remained strongly associated with increased 6-month mortality (adjusted Odds Ratio [aOR] 4.02, 95% CI 1.27–12.7, *P* = .02) (Figure 1). Among 86 participants with CMV viremia, 6-month mortality was similar among participants with CMV viremia < 1000 IU/mL versus CMV viremia ≥ 1000 IU/mL (27% [17/62] vs 21% [5/24], *P* = .53). There was no evidence of effect mediation by ART status or baseline CrAg status (likelihood ratio test *P* > .05).

**Figure 1.**
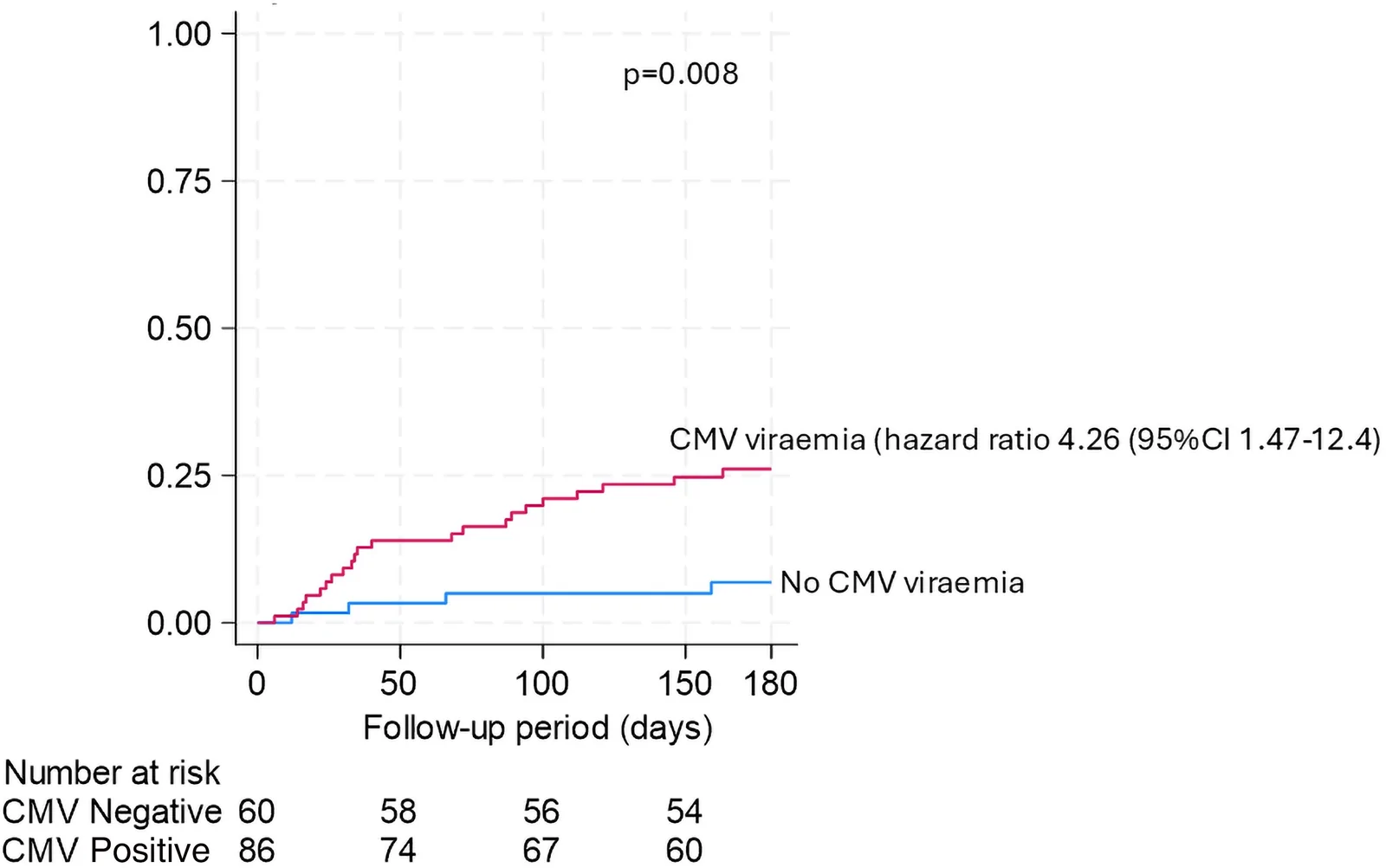
Cumulative 6-month mortality among 146 adults with advanced HIV disease, stratified by baseline cytomegalovirus (CMV) viremia status. Footnote: Participants with CMV viremia had greater than 4 times the hazard of all-cause 6-month mortality (unadjusted hazard ratio 4.26, 95% CI 1.47–12.4, *P* = .008) compared to those without CMV viremia. The number at risk table shows the number of participants remaining at risk at each time point; participants leave the risk set either because of death or censoring. There were 26 deaths overall (22/86 [26%] among participants with CMV viremia and 4/60 [7%] among those without CMV viremia); the remaining 6 participants who left the risk set were right-hand censored.

Overall, 12 cases (25%) of incident cryptococcal disease (ie, meningitis or cryptococcemia) were diagnosed during follow-up: 8 cases were among participants with CMV viremia versus 4 cases among participants without CMV viremia (*P* = .61). Overall, 40% (59/146) of participants had a history of TB disease or were diagnosed with TB disease during the 6-month study period; there was no evidence of an association between baseline CMV viremia status and diagnosis of TB disease at any time.

Minimally invasive autopsies were performed for 6 participants with CMV viremia, with confirmed evidence of CMV pneumonitis end-organ disease in 2 participants (Supplementary Table 2).

## DISCUSSION

Among this primarily outpatient cohort of adults with very AHD, CMV viremia was highly prevalent, occurring in over half of participants. Cytomegalovirus viremia was strongly associated with increased mortality, more than quadrupling the odds of death at 6 months. Only one-third of participants with CMV viremia for whom an MIA was performed had evidence of CMV end-organ disease, indicating that if the association between CMV viremia and mortality is causal, it is mediated in part by indirect effects of CMV rather than direct CMV end-organ disease.

Our prevalence estimate of 59% for CMV viremia among this primarily outpatient cohort with AHD is comparable to published literature [17], as is the strong and independent association with increased mortality. Our study adds to the considerable body of evidence demonstrating that CMV viremia is associated with adverse short- and medium-term outcomes in the context of AHD, including an increased risk of AIDS-defining events, tuberculosis, and death [9–11]. In the current study, we have demonstrated that the association between CMV viremia and mortality persists in the modern dolutegravir-based ART era and that participants with CMV viremia had 4 times the odds of 6-month mortality, irrespective of CD4 count or ART status. The 4-fold increased mortality risk associated with CMV viremia in this study is higher than anticipated based on our earlier work among hospitalized adults with AHD and cryptococcal meningitis, in which CMV viremia was associated with an ∼2-fold increased 10-week mortality [12]. Our findings suggest that the prognostic significance of CMV viremia may be more pronounced in outpatients with AHD than among seriously unwell hospitalized patients. The difference may be explained by the variable duration of follow-up across studies and/or by differential rates of (undiagnosed) opportunistic infections and/or immune reconstitution in outpatient versus inpatient AHD populations; further mechanistic studies are needed. The lack of observed association between baseline CMV viremia status and TB disease in this study is counter to several published studies that have reported on epidemiological, clinical, and immunological associations between CMV and *Mycobacterium tuberculosis* [7, 18]; given the relatively small sample size of our cohort, a type 2 statistical error should be considered.

The mechanisms for the observed associations between CMV viremia and death are poorly understood. It may be that within the context of AHD, CMV viremia is a bystander phenomenon, and its detection is a nonpathogenic marker of more severe immunosuppression [19]. The alternative hypothesis is that the immunomodulation induced by active CMV replication (including upregulation of type 1 interferons, such as interferon-alpha, and downregulation of type 2 interferons, such as interferon-gamma) impairs the host response to other opportunistic infections, thereby directly contributing to adverse outcomes [19].

Given this uncertainty, international HIV guidelines currently highlight the importance of early ART initiation rather than anti-CMV therapy for the management of CMV viremia [20–22], and there are currently no guidelines that recommend either primary prophylaxis or preemptive treatment for CMV viremia in the absence of CMV end-organ disease. However, in the context of AHD, where approximately 1 in 6 will die within 12 months of presentation to care, irrespective of ART and management of concurrent opportunistic infections, trials to evaluate whether preemptive treatment of CMV viremia can reduce mortality are required.

Our study has several limitations. First, the CICAS cohort study population was intentionally enriched for CrAg-positive participants. While we observed no statistically significant effect modification by baseline CrAg status, this enrichment likely led to overestimation of absolute mortality compared with an unselected AHD population. Second, MIAs were performed in only a small subset of deaths (n = 6); therefore, our estimate of the proportion of participants with CMV viremia who had end-organ disease should be interpreted with caution. While the sensitivity of MIAs specifically for detecting CMV end-tissue disease is unknown, concordance between MIAs and complete diagnostic autopsies for the detection of infectious diseases is reported to be high (79%) [23]. Third, follow-up was conducted primarily by telephone, without systematic microbiological testing for incident TB disease. As a result, some TB diagnoses were likely missed; however, we do not anticipate that this information bias differed across CMV viremia groups. Finally, while the directed acyclic graph as presented was developed following consultation with key experts in the field, we acknowledge that there remains considerable uncertainty with respect to the causal pathways underpinning these analyses. Specifically, without serial measurements of CMV and HIV viral loads over time, we were unable to determine the temporality between CMV viremia and HIV viral, and secondly we cannot discount the existence of unmeasured confounders at play within our analysis (eg, additional herpesvirus infections [24]) given the context of AHD. To ascertain whether the described associations are causal or not, interventional trials in the current era of HIV management are required. The recently started UK Medical Research Council-funded NIRVANA trial in South Africa and Uganda [25] is a phase 2b double-blinded, placebo-controlled randomized controlled trial to further evaluate the safety and virological efficacy of valganciclovir among hospitalized adults with AHD. Participants are randomized to receive valganciclovir for the management of CMV viremia or placebo for 28 days. The primary end-point is adverse events of special interest plus rehospitalization or death up to week 8.

In conclusion, among this primarily outpatient cohort of adults with AHD, CMV viremia occurred in 59% and was associated with greater than 4-fold increased risk of 6-month mortality. Cytomegalovirus viremia is a potentially modifiable determinant of mortality within this high-risk population.

## Supplementary Material

ofag615_Supplementary_Data

## Supplementary Data

Supplementary materials are available at *Open Forum Infectious Diseases* online. Consisting of data provided by the authors to benefit the reader, the posted materials are not copyedited and are the sole responsibility of the authors, so questions or comments should be addressed to the corresponding author.
