# Supplementary material for: Cytomegalovirus Viremia Is Strongly Associated With 6-Month Mortality Among Adults Living With Advanced HIV Disease: A Prospective Cohort Study in South Africa
Source: Open Forum Infect Dis. 2026 Sep 30;13(10):ofag615. doi: 10.1093/ofid/ofag615 (PMC13660684; doi:10.1093/ofid/ofag615)
Supplement: Supplementary file 1 [file ofag615_supplementary_data.docx]

**Supplementary tables and figures**

**Supplementary figure 1:** Directed Acyclic Graph (DAG)

This DAG presents the causal assumptions underpinning our analysis strategy. In the adjusted logistic regression models, age, sex, baseline CD4 count and antiretroviral therapy (ART) status at time of enrolment were included *a priori* as potential confounders of the association between CMV viraemia and six-month mortality. All other covariates were considered as potential confounders, except those co-variates which were considered potentially to lie on the causal pathway between CMV viraemia and mortality – Cryptococcal disease, tuberculosis disease, HIV viral load and C-reactive protein. Based on the available published literature, and consultation with experts in the field, we considered it biologically plausible that the association between CMV viraemia and mortality may be mediated by cryptococcal disease and/or tuberculosis disease and/or increased HIV viral load and/or inflammation as measured here by C-reactive protein due to the profound host immuno-modulation and immune dysregulation caused by active CMV replication. Therefore Cryptococcal disease, tuberculosis disease, HIV viral load and C-reactive protein were not included as confounders in the adjusted logistic regression models. We recognise however, that c**onsiderable uncertainty remains regarding the potentially causal pathways between CMV viraemia and mortality, including the temporality of covariates including HIV viral load.**

**Supplementary table 1: Baseline characteristics and outcomes of 146 adults with advanced HIV disease stratified by baseline CMV viraemia status.**

| Variable | Total cohort  N (%) or  median (IQR) | CMV viraemia  N (%) or  median (IQR) | No CMV viraemia  N (%) or  median (IQR) | P value |
| --- | --- | --- | --- | --- |
| Total | 146 | 86 (58.9) | 60 (41.1) |  |
| Demographics: |  |  |  |  |
| Male | 73 (50.0) | 43 (50.0) | 30 (50.0) | 1.00 |
| Age (years) | 39 (33-48) | 39 (34-48) | 41 (32-46) | 0.55 |
| Baseline: |  |  |  |  |
| Receiving ART at baseline | 29 (19.9) | 21 (24.4) | 8 (13.3) | 0.10 |
| Baseline CD4 (cells/μL) | 32 (10-60) | 26 (9-49) | 38 (18-64) | **0.04** |
| Baseline C-Reactive Protein | 53 (15-116) | 53  (15.5-120.5) | 58  (17.5-104.5) | 0.82 |
| Baseline serum CrAg positive | 48 (32.9) | 29 (33.7) | 19 (31.7) | 0.80 |
| Baseline urinary TB-LAM positive | 28 (20.3) | 18 (21.7) | 10 (18.2) | 0.62 |
| Active TB at baseline | 55 (37.7) | 31 (36.1) | 24 (40.0) | 0.63 |
| Outcomes: |  |  |  |  |
| Any TB disease (baseline or incident) | 59 (40.4) | 32 (37.2) | 27 (45.0) | 0.34 |
| Cryptococcal meningitis or cryptococcaemia | 12 (25.0) | 8 (27.8) | 4 (21.0) | 0.61 |
| 6-month mortality | 26 (17.8) | 22 (25.6) | 4 (6.7) | **0.003** |
| p value from chi squared test for categorical variables, and t-tests for continuous variables (normal distribution) or Mann-Whitney for comparing medians.  Abbreviations: IQR = interquartile range, CMV = cytomegalovirus, ART = Anti-retroviral treatment, CrAg = Cryptococcal antigen, TB-LAM = Tuberculous Lipoarabinomannan, TB = Tuberculosis  Baseline CMV viraemia was detected in 58.9% (86/146, 95%CI 50-67%), with a median viral load of 366 (IQR 171-1,187) IU/mL.  HIV viral load measurements were not routinely conducted as part of the study, of 18 participants with available HIV viral load data, the median HIV viral load was 339, 137 (IQR 87,100 - 1,390,000) copies/ml.  Overall, 18% (26/146) of participants required admission to hospital for acute medical care at time of enrolment; this proportion was higher amongst participants with CMV viraemia (26%, 22/86) vs. amongst participants without CMV viraemia (7%, 4/60), p=0.004. | | | | |

**Supplementary table 2: Baseline demographics, clinical details and CMV testing results from two study participants who underwent minimally invasive autopsy with confirmed CMV end organ disease.**

| **Baseline clinical details** | **CD4 cell count (cells/μL)** | **Baseline CrAg status** | **ART status** | **Baseline CMV VL (IU/ml)** | **Time to death (days)** | **Histological evidence of CMV end organ disease at minimally invasive autopsy (MIA).** | **MIA evidence of co-prevalent AIDS defining illness** |
| --- | --- | --- | --- | --- | --- | --- | --- |
| 42M | 19 | Negative | ART naïve | 311 | 24 | CMV pneumonitis: Enlarged cell (arrow A), with CMV confirmed by immunohistochemistry (arrow B).   | Pneumocystis pneumonia |
| 25F | 12 | Positive | ART naïve | 285,980 | 6 | CMV pneumonitis: CMV inclusion body (arrow A), CMV confirmed by immunohistochemistry (not shown).   | Kaposi’s sarcoma |
| Abbreviations: CrAg = Cryptococcal antigen, ART = Anti-retroviral treatment, CMV = cytomegalovirus, qPCR = quantitative polymerase chain reaction, VL = viral load, MIA = minimally invasive autopsy. | | | | | | | |

**Supplementary methods**

**Supplementary methods 1**

**Baseline opportunistic infection screening provided for 146 adults with advanced HIV disease**

| Study time point | Sample type | Microbiological testing |
| --- | --- | --- |
| Enrolment | Blood | Prolonged (21days) fungal culture  C-reactive protein (CRP) testing |
| Enrolment | Pre- and/or post induction sputum | Auramine staining and Microscopy  Liquid culture [BACTEC MGIT, Becton Dickinson, Franklin Lakes, NJ]  Molecular testing [Xpert MTB/RIF, Cepheid, Sunnyvale, CA] |
| Enrolment | Urine | Tuberculosis-lipoarabinomannan (Determine TB LAM, Alere, Waltham, MA)  Xpert MTB/RIF Ultra testing  Histoplasma antigen enzyme immunoassay (IMMY) |

**Supplementary methods 2**

MIA procedures were similar to those described in previous MIA studies^14,15^. An initial external examination of the body was performed, and organs were located using external anatomical landmarks or ultrasound. Standardized multiple core biopsy samples were obtained from lungs, liver, spleen, and kidneys using a 14-gauge core biopsy needle. CSF from the cisterna magna (suboccipital approach) and blood from the subclavian vein were aspirated using 18-gauge needles and syringes. Bronchoalveolar lavage (BAL) was performed using normal saline delivered through a nasogastric tube into the bronchi through a tracheal incision. If abnormalities were clinically detected, aspirates of pleural and pericardial fluid and punch biopsies of any skin lesions (excluding on the face or neck) were taken. Tissue cores were preserved in formalin, embedded in paraffin, and stained with hematoxylin-eosin. Additional stains to assess for the presence of acid-fast bacilli (Ziehl-Neelsen), bacteria (Gram stain), and fungi (Grocott’s methenamine silver) were performed if any features of inflammation were seen. Cytomegalovirus immunoperoxidase staining was performed when characteristic viral inclusions were noted. Tissue cores, CSF, BAL, and pericardial fluid underwent bacterial, fungal, and mycobacterial cultures; bacterial and viral multiplex polymerase chain reaction; and Xpert MTB/RIF testing. Plasma, CSF, and BAL fluid were tested using the IMMY CrAg LFA.Immediate and contributing causes of death were attributed by a panel of 6 infectious diseases physicians and pathologists at the University of the Witwatersrand Department of Anatomical Pathology.
